# Enhanced electrical properties of vertically aligned carbon nanotube-epoxy nanocomposites with high packing density

**DOI:** 10.1186/1556-276X-7-630

**Published:** 2012-11-16

**Authors:** Tewfik Souier, Sergio Santos, Amal Al Ghaferi, Marco Stefancich, Matteo Chiesa

**Affiliations:** 1 Laboratory of Energy and Nanosciences, Masdar Institute of Science and Technology, Abu Dhabi, PO Box 54224, United Arab Emirates

**Keywords:** Carbon nanotubes, Nanocomposites, Polymers, Electrical conductivity, Conductive AFM

## Abstract

During their synthesis, multi-walled carbon nanotubes can be aligned and impregnated in a polymer matrix to form an electrically conductive and flexible nanocomposite with high backing density. The material exhibits the highest reported electrical conductivity of CNT-epoxy composites (350 S/m). Here, we show how conductive atomic force microscopy can be used to study the electrical transport mechanism in order to explain the enhanced electrical properties of the composite. The high spatial resolution and versatility of the technique allows us to further decouple the two main contributions to the electrical transport: (1) the intrinsic resistance of the tube and (2) the tunneling resistance due to nanoscale gaps occurring between the epoxy-coated tubes along the composite. The results show that the material behaves as a conductive polymer, and the electrical transport is governed by electron tunneling at interconnecting CNT-polymer junctions. We also point out the theoretical formulation of the nanoscale electrical transport between the AFM tip and the sample in order to derive both the composite conductivity and the CNT intrinsic properties. The enhanced electrical properties of the composite are attributed to high degree of alignment, the CNT purity, and the large tube diameter which lead to low junction resistance. By controlling the tube diameter and using other polymers, the nanocomposite electrical conductivity can be improved.

## Background

Carbon nanotube polymer nanocomposites (CNT-PNCs) have been extensively investigated due to their enhanced properties [1-3]. The advantages of the composite include (1) their superior electrical and thermal conductivities combined with high mechanical strength and (2) retention of the mechanical properties of the polymer matrix such as flexibility and processibility. A nanocomposite with this set of characteristics has the potential to serve as a low cost and flexible conductor in the electronics and aerospace industries, as well as in actuators, nanoelectrodes, and sensors [4-6].

The electrical conductivity of the composite of metallic particles/fibers in an insulating matrix is well described by the percolation theory. The use of high aspect ratio filler, such as CNT, results in a percolation threshold (minimum amount of filler to create a percolation path to electrical current) much lower than the spherical-particle fillers [7]. However, the measured electrical conductivities of CNT-based polymer composites range from 10^−5^ to 480 S/m at or above the percolation threshold [8-10]. This large range in the reported data may reflect the complex nature of the problem. A correlation between the transport properties of the composite and its structural characteristics is challenging yet important to decouple the effects of different contributions. A deep understanding of the transport phenomena in CNT-PNCs requires a full description of the nanostructure. Structural studies commonly rely on scanning and transmission electron microscopy (SEM/TEM) data. Nevertheless, undesired structural changes can occur in the characterization process [11-13]. Alternatively, scanning probe microscopy and mainly conductive atomic force microscopy (C-AFM) are used to characterize carbon nanofiber embedded in SiO_2_ to interconnect in integrated circuits [14,15] and randomly dispersed multi-walled carbon nanofiber polymer nanocomposites (R-CNT-PNCs) [16]. The advantage of this technique is to probe simultaneously the structural and electrical properties with nanoscale resolution.

In this letter, we employ a recently developed catalytic chemical vapor deposition (CCVD) technique that yields controlled-morphology aligned carbon nanotube (A-CNT). The resulting CNT carpet is filled with epoxy polymer to fabricate the aligned carbon nanotube-based polymer nanocomposite (A-CNT-PNCs). The composite exhibits the highest electrical conductivity reported in the field (CNT-epoxy composites). Structural and electrical characterization is needed to understand the enhanced electrical properties and the transport mechanism. The nanostructure of the nanocomposite is revealed by SEM and C-AFM techniques. The structural study reveals that the composite material exhibits a high CNT filling fraction up to 15% higher than the maximum filling fraction (4%) obtained on R-CNT-PNCs [17]. We further employ C-AFM to probe the morphology-dependent electrical transport properties of the composite at the nanoscale. The C-AFM setup used in this study allows current measurements up to 10 orders of magnitudes. The versatility of the instrument allows us to decouple and distinguish between the intrinsic resistance of the CNTs and the contact resistance with the electrode (conductive AFM tips). This approach is shown to yield the intrinsic electrical properties of the multi-walled CNTs as well as the volume conductivity of the A-CNT-PNCs.

## Methods

The nanotubes used here are multi-walled (MWCNT) and have been grown vertically on quartz by catalytic chemical vapor deposition as described elsewhere [18,19]. The resultant vertically aligned carbon nanotube (VACNT) carpet was annealed at 2,000°C for 2 h in argon to produce high-purity MWCNT. The CNTs were then impregnated by epoxy and later digressed in order to ensure that the polymer properly infiltrates between the CNTs. The samples were cured at 60°C for 4 days, grounded with SiC paper to a grit of 4,000, and polished with 1- and 0.25-μm diamond paste. At the end of each step, the VACNT carpets have been characterized by SEM and TEM. The final composite length was found to be 540 μm. The structural and electrical characterization was also carried out with an MFP-3D AFM from Asylum Research (Santa Barbara, CA, USA). Unlike the previous study using C-AFM, our instrument allows measurement of current from 1 pA up to 1 mA, thus, 10 orders of magnitude [20-22]. Silicon tips with two different conductive coatings were employed: (1) AC240TM platinum-coated tip from Asylum Research® with 30-nm apex radius and (2) n-doped diamond-coated tip from NT-MDT® (Zelenograd, Moscow, Russia), with 70-nm apex radius [see Additional file 1.

## Results and discussion

**Figure 1:**
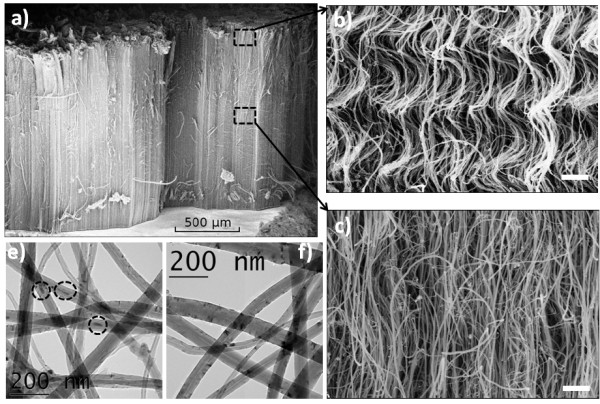
**SEM and TEM images.** (**a**) SEM image of the CNT carpet at low magnification. SEM images at high magnification showing (**b**) the entangled CNT structure on the top of the carpet and (**c**) the aligned structure along the carpet. TEM images of the MWCNTs (**d**) before and (**e**) after annealing.

The VACNT carpets, extracted after CCVD growth, are shown to be 1.45-mm thick (Figure 1a). The MWCNTs are well aligned within the carpet thickness (Figure 1c) with the exception of the entangled structure on the top (Figure 1b). The SEM image (Figure 1c) shows that the MWCNTs (in the middle of the carpet) are quite straight along the carpet but with a possible point of contact between them. Moreover, short nanotubes are found to be aligned in the transverse direction forming T-junctions with aligned CNTs. This area of VACNT carpet is the one used to form the polymer nanocomposite. The description above is one of the key results for the interpretation of the electrical C-AFM data.

In order to perform the TEM structural analysis of CNTs, a small piece of the carpet (before and after the annealing process) was dispersed in an ethanol solution (Figure 1d,e). Inside the core of the as-grown CNT, some iron particles were encapsulated. The annealing process does not involve any modification of the CNT diameter, but it almost completely remove the iron particles. After being annealed, the NTs' extremities are found to be open. These observations suggest a high-purity and crystalline CNTs which enhanced their electrical properties [23,24].

The distribution of external (and internal) CNT diameters is found to resemble a Gaussian. The statistical analysis shows that the average external diameter of the tubes is 48 nm, and the internal diameter is 7 nm.

**Figure 2:**
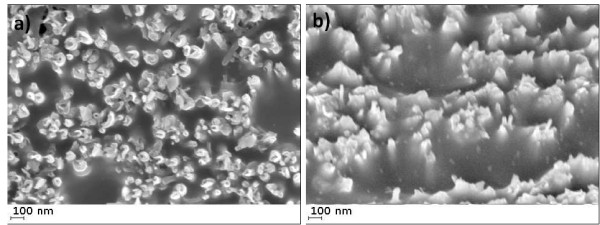
**SEM images of the as-polished composite surface.** (**a**) Top view and (**b**) when the sample is tilted by 42°.

Particular care is paid in our analysis to the quality of the interface, i.e., CNT external contact, this being a main mechanism affecting electrical conductivity measurements. In Figure 2a,b, the SEM images of the as-polished composite surface performed with secondary electron detector in two cases: where (1) the electron beam is perpendicular to the sample surface (Figure 2a) and where (2) the sample is tilted by 42° (Figure 2b) are shown. The CNTs appear open-tipped, and some of them are not fully circular; the missing parts of CNT tips are probably removed during the mechanical polishing procedure. The diameter of individual CNTs observed by SEM confirms the observation previously made by TEM. However, the SEM images reveal the presence of nano-objects with diameters greater than 150 nm. This could be interpreted as some of the CNTs grouping in bundles. On tilted views, the CNTs are protruding from the polymer matrix by a few tens of nanometers. The protruding length of CNTs can significantly affect the electrical interface resistance and can be estimated using AFM. For this purpose, AFM experiments have been carried out using diamond-coated (and platinum-coated) tips in the contact mode. The height images reveal the existence of topographic peaks that can be identified as individual CNTs or CNT bundles [see Additional file 1]. It may be noted that the diameters match well the diameters of CNTs observed by SEM. The surface roughness analysis confirms that the CNTs protrude a few tens of nanometers (and up to 80 nm) above the polymer matrix (average protrusion ≈ 48 nm).

Since the electrical properties of the composite depend strongly on the content and distribution of CNT fibers, a statistical analysis has been carried out first on several SEM images. The results show that the density of CNTs is 4.3 ± 1.5 10^13^ CNT/m^2^, and their average surface fraction is 15%. This is far superior to the volume fractions measured on R-CNT-PNCs.

**Figure 3:**
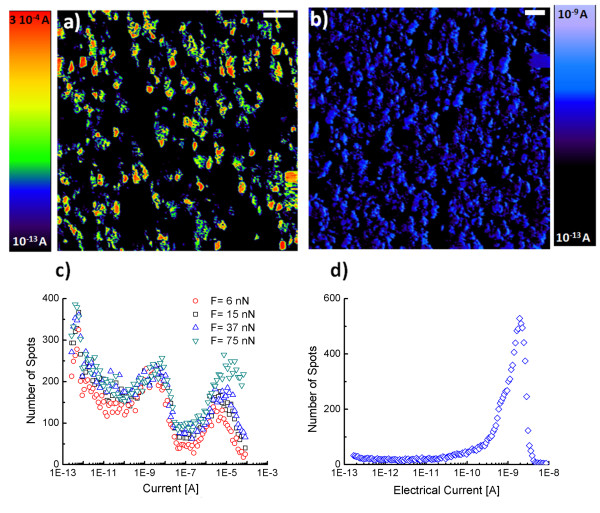
**The CS-AFM images of the as-polished nanocomposite.** (**a**) With Pt-coated tip and (**b**) with doped diamond-coated tip; (**c**) and (**d**) show the histograms of current extracted from the images (**a**) and (**b**), respectively. The histogram (**c**) is performed at various tip-sample forces in order to decouple the tip-sample contact resistance from the nanotube and composite resistance.

The AFM height and friction (lateral force) images have been used, but they lead to an underestimation of the CNT filling fraction [see Additional file 1]. A way to circumvent this problem is to employ C-AFM; high resolution current maps may be obtained with low load (6 nN) by platinum-coated tip. The spatial resolution estimated from measuring the contact area is less than 4 nm. According to TEM analysis of the CNTs radii, all the tubes should be resolved. The current map (Figure 3a) reveals highly resistive regions (black) corresponding to the polymer matrix and highly conductive regions (red, green, and blue) corresponding to CNTs. The average diameter of the conductive spot is approximately 50 nm, which is in good agreement with the TEM analysis. Furthermore, image analysis on several current maps give a CNT density ≈ 4.8 ± 0.5 10^13^ CNT/m^2^. This agrees with the value obtained from the SEM analysis. Moreover, in the high-magnification current map, the open top CNT structure may be observed. This is again in agreement with the SEM observations [see Additional file 1].

The structural properties of the composite material, i.e., the filling fraction, structural defects inside the CNT core, the aspect ratio, and the alignment and percolation of the CNTs strongly affect the electrical properties of individual nanotubes as well as the overall conductivity of the composite. After a full characterization by means of various techniques, the study can be directed towards the characterization of the electrical transport of the system, first at the macroscale and then at the nanoscale. For this purpose, gold electrodes (approximately 300-μm wide and 100-nm thick) were evaporated on both sides of the composite sample in order to perform two and four terminal electrical measurements. The electrical conductance is found to be on the order of 0.08 S, both in the vertical direction. The electrical conductivity of the composite is estimated using a simple Ohm's law, $\rho_{\text{Comp}}=\frac{RL}{W^{2}}\text{,}$ where *R* is the measured resistance; *W*, the width of the gold electrode; and *L*, the composite length. In this manner, we obtain a value of conductivity of 350 S/m. This value is the highest electrical conductivity of epoxy-CNT composites and is among the highest reported values in CNT-polymer composites [17,25].

In order to understand the macroscopic data, the C-AFM is employed. By using the nanosized conductive tip, reduction in the contact resistance should follow. In turn, this allows the electrical conductivity of the composite to be determined with improved accuracy. Moreover, in C-AFM, the nanoscale nature of the measurement allows for the intrinsic electrical conductivity of single CNTs to be determined.

In the Figure 3a,b, the current C-AFM images obtained under +2 V sample bias and using platinum- and diamond-coated tips, respectively, are shown. The current images remain similar under high and low voltages (from 0.1 to 2 V) and under positive and negative voltages. The images show large dark areas with very low current (0.1 to 1 pA) corresponding to high resistance values (*R* = *V* / *I*) in the range of 1 to 10 TΩ. These areas correspond to the epoxy matrix. These have been reported to have high electrical resistivity in the range of 10^15^ Ω·m [26]. The same images show colored areas (red, green, and black); these are identified here as the CNTs. The histogram of the electrical current extracted from the current maps show peaks of conductive spots. In the case of the diamond tip, one peak is observed with a mean value of resistance ranging from 1 up to 50 GΩ (Figure 3d). These scattered data are attributed to different properties of the doped diamond coating. Indeed, the intrinsic resistances of these tips (from the same wafer) vary from 1 to 100 MΩ. This could be a result of different manufacturing processes or doping levels.

On the other hand, more reproducible data are obtained using coated platinum tips. A specific procedure [20] that provides a reproducible data is used in this study. We point out the stability of the current level in C-AFM images during the time of the experiment (several hours). In Figure 3c, the histogram of current reveals the existences of two log-normal distributions. The first distribution (*D*1) corresponds to high electrical resistances (10 MΩ to 10 GΩ). The second distribution (*D*2) corresponds to low electrical resistances (6 kΩ to 1 MΩ).

In order to understand the different electrical properties of the CNT using the two conductive tips, the intrinsic resistivity of each tip is estimated on a highly conductive gold film (approximately 100-nm thick): *R*_Pt_ approximately 10^2^ Ω and *R*_Diamond_ > 10^6^ Ω. We note that the measured resistances of gold film using certain diamond tips can reach values in the order of gigaohms which suggests a creation of a Schottky barrier at the tip-substrate interface. A similar behavior could be suggested to interpret what was observed in the A-CNT-PNC sample; assuming the MWCNT (average diameter approximately 48 nm) as a metallic conductor with an electron work function of 5 eV [27] and assuming the diamond tip as highly doped n-type semiconductor with affinity *χ* = 2.7 eV and band gap of 5.45 eV [28], a Schottky barrier forms at the nanocontact CNT tip with a height of approximately 2.3 eV. Thus, the low current measured on the composite surface with a diamond-coated tip could be associated to a high contact resistance *R*_tip-CNT_, and thus, such a tip cannot be used to characterize CNT-based materials.

The explanation above holds for the metallic shells of the MWCNT. However, it is known that depending on the chirality, the shells composing the MWCNT can be metallic, semimetallic, or semiconductor. For semiconducting shells, the energy gap depends upon the reciprocal nanotube diameter *d*, *E*_g_ = 2*γ*_0_*a*_C-C_/*d*, where a_C − C_ = 0.142 nm is the C-C distance and *γ*_0_ = 2.45 eV [29]. At room temperature (*T* = 300 K), the energy gap smaller than *k*_B_*T* = 0.0258*eV* is smeared by temperature. Accordingly, the shell with a diameter higher than 25 nm can be considered as conductor at room temperature. According to TEM analysis, this covers a large range of considered MWCNT. The rest of the shells can be considered as semimetallic since the largest expected gap for 2.5-nm diameter shells is about 270 meV.

In the case of platinum-coated tip, on the other hand, the resistance of the tip *R*_tip_ may be neglected, and thus, the tip may be used to quantify the electrical properties of the composite if the tip-sample contact resistance *R*_tip-CNT_ is also negligible. Unfortunately, the metal-CNT contact is a complex problem which cannot be explained with the Schottky theory but instead involves the pinning of the Fermi level and the adhesion between the metal and the CNT [30]. Platinum exhibits a quite high contact resistance with the CNT in comparison to palladium. This has been attributed to the de-wetting property of platinum on the CNT, while the palladium wets the CNT well and leads to a lower contact resistance [31]. Under these assumptions, the electrical transport is controlled by a tunneling mechanism, and the contact should not be considered as ohmic. However, local *IV* curves performed with platinum tip exhibit both non-ohmic and ohmic behaviors. The ohmic *IV* curve corresponds to an ohmic contact probably due to a good mechanical contact with metallic shells, and the non-ohmic corresponds to a formation of Schottky or tunneling barrier with semimetallic shells of MWCNT (see the description above). However, in the two situations, the contact resistance is dependent on the tip-sample contact size *R*_tip-CNT_ = *f* (*a*). The global electrical resistance (*R* = *V* / *I*) measured by C-AFM can be written as follows: *R* = *R*_tip-CNT_ + *R*_comp_ = *f*(*a*) + *R*_comp_, where *a* is the tip-sample contact radius, and *R*_comp_ is the composite resistance.

In Figure 3d, the histogram of C-AFM current is shown. The current (or resistance) is measured at various loads (6 up to 75 nN). These changes in force result in variations in the contact radius *a* ranging from 2.5 to 5 nm [see Additional file 1]. From the figure, it may be concluded that the second distribution (*D*2) of current is strongly affected by the applied loads and, thus, depends on the interaction forces between the tip and the sample. However, the first distribution (*D*1) does not change significantly with varying load. Therefore, we deduce that the contact resistance *R*_tip-CNT_ is in the range of the resistances of distribution (*D*2), i.e., 6 kΩ to 1 MΩ. On the other hand, the resistances of the distribution (*D*1), i.e., 10 MΩ to 10 GΩ, are related to the electrical transport mechanism inside the composite.

The contact resistance of T-junctions between single-walled carbon nanotubes (SWCNTs) is in the range of 100 to 400 kΩ if the SWCNTs are both metallic or semiconductor and 2 orders of magnitude higher for metal/semiconducting junctions [32,33]. These values are not in the range of measured composite resistances of 10 MΩ to 10 GΩ. The plausible reason of such large resistances is the tunneling junctions that may occur between the CNTs which involve a contribution from the epoxy inter-CNT layer. This assumption is supported by the structural characterization of CNT carpet by SEM where the point of contact between CNT and the existence of horizontal small CNT are observed (Figure 1c). If we assume that a few atomic layers cover the external shells of MWCNTs, then the tunneling resistance could reach the 10 MΩ to 10 GΩ range. The spots with such resistance values, i.e., green and blue spots in the conductive image (Figure 3a), correspond to either CNTs with small diameters or the outmost shells of the CNTs with the larger diameters. It is obvious that the outmost shells of MWCNTs, covered with an epoxy layer, are sensitive to tunneling via epoxy and adjacent CNTs. Moreover, the presence of short perpendicular CNTs that connect several aligned CNTs increases the probability of the creation of these junctions.

We conclude here that C-AFM allows separation between two resistances *R*_1_ and *R*_2_ of the two distributions *D*1 and *D*2. The measured resistance by C-AFM can be written as follows: *R* = *R*_1_ + *R*_2_, with *R*_2_ = *R*_tip − CNT_ + *R*_MW_ and *R*_2_ = *R*_T_ as the tunneling resistance between CNT-epoxy-CNT junctions.

We point out here that the ability of isolating these different contributions to the electrical transport within the composite by means of C-AFM is due to the high lateral resolution of the technique. That is, C-AFM permits studying the electrical properties of only a few shells within a MWCNT. For example, the tip-sample contact diameter is in the range of 2.5 to 6 nm [see Additional file 1] which is much smaller than the diameter of the MWCNT (average value of 48 nm). Accordingly, electrical transport in C-AFM involves only a few shells of the MWCNTs. If we take a contact size of 2*a* = 2.5 nm and take into account inter-wall distances of *δ* = 0.34 nm, the number of shells that participate in conduction is 8 (*N*_Shell_ = 1 + [2*a*/*δ*]). Thus, it is possible to probe separately the electrical properties of internal and external shells of MWCNT.

One should recall however that the electrical transport within the A-CNT-PNCs is a complex phenomenon. On the other hand, under certain assumptions, the electrical conductivity of MWCNTs and the composite can be estimated. We assume the following: (1) the contact between the AFM tip and the CNT is ohmic; this assumption implies that one can use Sharvin's formula [34] for the contact resistance *R*_tip-CNT_. (2) The intrinsic resistance of the nanotube *R*_MW_ may be expressed simply through Ohm's law involving the CNT cross-section *A*_CNT_. With these two assumptions, the electrical resistivity of the nanotube may be estimated.

$$\left\{ \begin{array}{l} R_{\text{tip-CNT}}=\frac{4}{3}\frac{\rho \lambda }{\pi a^{2}}\\ R_{\text{MW}}=\frac{\rho L}{A_{\text{MW}}} \end{array}\text{,}\right.$$

where *L*, *A*_MW_, and *λ* are the CNT length, cross section, and electron mean free path, respectively. This formula is valid only in the range of resistances *R*_2_ within the second distribution (*D*2). The cross-sectional area of the nanotube can be calculated as follows: *A*_CNT_ = *π*/4*D*^2^ − (*D* − *Nδ*)^2^ where *D* is the difference between the external and internal tube diameter, and *N* is the number of shells composing the MWCNT.

The quantity *ρ* × *λ* is usually considered as constant for most metal and is in the order of 10^−15^ Ωm^2^[35]. This cannot be used in case of carbon nanotube which is a one-dimensional material. However, for a SWCNT, the product of its linear resistance *r* and its electron mean free path *λ* corresponds to the quantum ballistic limit *r* × *λ* = 6,454 Ω [36]. The electrical resistivity of MWCNT with *N* shells can be given by *ρ* = *r* × *A*_CNT_ / *N*.

The resistivity of MWCNTs, the average linear resistivity of shells, and the electron mean free path are estimated by using the mean value of the measured resistances *R*_2_ and for different values of tip-CNT contact size *a*. We obtain *ρ* approximately 10^−6^ Ω·m, *r* approximately 10 kΩ/μm, and *λ approximately 480 nm*. These values are in the range of the referenced value of high-purity carbon nanotubes [37-39]. This result shows that the composite processing does not affect the electrical properties of individual MWCNT.

The composite volume resistance may be considered as the sum of the intrinsic resistance of the tube and the CNT-epoxy-CNT tunneling resistance and can be expressed as follows: R_comp_ = R_MW_ + R_T_ ~ R_T_. Indeed, *R*_MW_ is found to be in the order of 100 kΩ, while the tunneling resistance *R*_T_ is 3 orders of magnitude higher. Thus, by using Ohm's law, the volume conductivity of the composite is estimated to be in the range of 100 to 900 S/m with an average value of 350 S/m. This value is in the range of the macroscopic value obtained by four probe measurements.

We would like to point out that both the electrical conductivity of single CNT and the composite volume conductivity are calculated by (1) assuming CNT with a diameter of 48 nm (mean value according to TEM images) and (2) taking a value of resistance at the center of the measured log-normal distributions.

Finally, we would like to comment on the distribution of the resistances which is found to be log-normal, i.e., Log(*R*) spread as a Gaussian distribution. Intuitively, this distribution is related to the Gaussian distribution of the external and internal diameters of the tubes measured by TEM [see Additional file 1] and, more precisely, to the number of the shells of MWCNT. In case of the second distribution 2 (6 kΩ to 1 MΩ), the resistance scales with the tube cross-section *A*_CNT_ = π/4[*D*^2^−(*D*−*Nδ*)^2^]. Assuming the same electrical conductivity of the tube 10 to 6 Ω·m, we obtain an intrinsic resistance of 7 kΩ for a 110-nm CNT diameter and 800 kΩ for a 10-nm CNT diameter. This matches extremely well to the limits of the distribution of resistances *D*2. In case of the first distribution *D*1, the tunneling resistance scales with the tunneling area at interconnecting CNTs which could be expressed as *A*_T_ approximately *D*^2^, where *D* is the external tube diameter. However, assuming a volume conductivity of 350 S/m, we obtain by simply using Ohm's law a resistance of 100 MΩ and 150 GΩ for CNT diameters of 110 and 10 nm, respectively. We conclude that the Gaussian distributions (*D*1) and (*D*2) of Log(*R*) are related to the Gaussian distribution of MWCNT diameters. Moreover, in order to improve the composite electrical conductivity, low tunneling resistances are needed, and this can be obtained by simply manufacturing MWCNT with high diameters.

## Conclusions

In summary, the structural and electrical characterization of the vertically aligned multi-walled CNT arrays embedded in epoxy resin has been performed by means of scanning and transmission electron microscopes coupled with conductive atomic force microscopy C-AFM. The composite exhibits the highest reported electrical conductivity measured at macro- and nanoscales. The diameter and the density of CNTs obtained by C-AFM agree well with those obtained by TEM and SEM imaging. Moreover, the C-AFM is found to be a robust technique to probe the conductance of CNT with a lateral resolution of ≈4 nm. This implies that individual characterization of each CNT can be achieved. Thanks to the capability of C-AFM pre-amplifier, the current maps reveal higher contrast than the height and friction maps, and the measured current ranges from tenths of pico-amps up to hundreds of micro-amps. Indeed, as stated above, the characterization technique allows sensing current (or electrical resistance) variation up to 10 orders of magnitudes. The map of the local resistance (*R* = *V* / *I*) exhibits a bimodal log-normal distribution; the first one, centered at a high resistance of 6 × 10^8^ Ω, is attributed to tunneling transport within CNT-polymer-CNT junctions. The second one, observed at low resistances of 10^5^ Ω, is attributed to the intrinsic CNT conductivity and tip-sample contact resistances. A simple model has been employed here to disentangle these different contributions. This has allowed estimating both the electrical conductivity of individual CNTs and also the volume conductivity of the nanocomposite. The information obtained from the investigation presented in this work has the potential to play a significant role in the study and establishment of percolation-based models for the electrical transport in aligned multi-wall CNT arrays. Such model may provide the necessary understanding to significantly improve the material processing of CNT-based conductive polymer nanocomposites.

## Competing interests

The authors declare that they have no competing interests.

## Authors' contributions

TS grew the samples, performed the AFM measurements and the theoretical calculations, and wrote the manuscript. SS took part in the interpretation of the AFM data and contributed to the editing work. AG read the article and contributed to the article improvement. MS participated in the elaboration of the theoretical model and the article improvement. MC led the study and the team that performed the investigation, participated in the conception of the project, and edited the manuscript. All authors read and approved the final manuscript.

## Supplementary Material

Additional file 1 Further details on material fabrication (Figure S1), the conductive AFM setup (Figure S2), AFM tip-sample interactions (S3), AFM height and friction images (Figure S4), and the correlation between SEM and C-AFM imaging (Figure S5) are given. Click here for file
