# Supplementary material for: Enhanced electrical properties of vertically aligned carbon nanotube-epoxy nanocomposites with high packing density
Source: Nanoscale Res Lett. 2012 Nov 16;7(1):630. doi: 10.1186/1556-276X-7-630 (PMC3511210; doi:10.1186/1556-276X-7-630)
Supplement: Additional file 1 — Further details on material fabrication (Figure S1), the conductive AFM setup (Figure S2), AFM tip-sample interactions (S3), AFM height and friction images (Figure S4), and the correlation between SEM and C-AFM imaging (Figure S5) are given. [file 1556-276X-7-630-S1.doc]

# “Enhanced Electrical Properties of Vertically Aligned Carbon Nanotubes−Epoxy Nanocomposites with High Packing Density”

by Tewfik Souier*, Sergio Santos, Amal Al Ghaferi, Marco stefancich, Matteo Chiesa

**Supporting Information**

Further details on material fabrication (S1), the conductive AFM set-up (Figure S2), AFM tip-CNT interactions (S3), AFM height and friction images (Figure S4), and the correlation between SEM and C-AFM imaging (Figure S5) are given.

**Material fabrication (Figure S1),**

The polymer used for the encapsulation is a mixture composed of 47 wt% of Epon (*Epon 812*), 52.2 w% of MNA (*Methyl - 5 - norbornene - 2,3 - dicarboxylic anhydride -* C10H10O3) and 0.8 wt% of DMP30 (*2,4,6 - Tris (dimethylaminomethyl) phenol -* [(CH3)2NCH2]3C6H2OH).

**Conductive AFM set-up (Figure S2),**

The conductive tips used in the study are : 1) AC240TM platinum coated tip from Asylum Research®, (spring constant ~ 2 N/m and the apex radius ~ 30 nm); 2) n-doped diamond coated tip from NT-MDT®, (spring constant ~ 30 N/m and the apex radius ~ 70 nm), the coating is doped with nitrogen and the film resistivity is 0.5 to 1 Ω cm.

**S3: AFM tip-CNT interactions**

In order to probe the structural and electrical properties of individual CNT, high spatial resolution is needed. Thus, the tip-sample contact area is estimated by means of Hertz[[1]](#footnote-2) mechanics: $a=\left(3R_{tip}P/4E^{*}\right)^{1/3}$ where *P* is the tip-sample contact force, R*_tip_*=30 nm is the tip radius, and *E^*^* is the effective elastic modulus given by: $1/E^{*}=\left(1-\upsilon_{tip}^{2}\right)/E_{tip}+\left(1-\upsilon_{s}^{2}\right)/E_{s}$ where *υ_tip_*=*υ_s_*=0.33 is the poison’s ratio of the tip and the sample, *E_s_* is the Young modulus of the sample (1Tera Pa for CNT axial moduli and 3.5 GPa for epoxy) and E_tip_ is the Young modulus of the tip (1.2 Tera Pa for diamond and 168 GPa: platinum). The tip-sample loads are in the range of 6 nN to 100 nN. Higher loads of few 100 nN are necessary to obtain resolved friction images using diamond tip. Using these values, the diameter of the contact ranges from 2.5 – 6 nm on CNT and from 10 – 30 nm on epoxy. By using diamond tip with large forces (few 100 nN) the diameter of contact area on top of CNT can be higher than 15 nm. The AFM indentations δ are also estimated using the following formulas:$\delta=a^{2}/r_{tip}$. The indentations are estimated to be less than 0.5 nm on CNT but ranges from 1.5 – 7 nm on the Epoxy.

According to these estimations and to the TEM analysis of the diameter of the tube (see below), all the CNT can be identified in AFM images in the experimental conditions with the exception of using diamond tip with high forces (few 100 nN). Moreover, the indentations on the epoxy are sufficiently high to remove any epoxy layer covering the tip of CNT during the AFM scans.

**AFM height and friction images (Figure S4),**

Figure: High resolution AFM images a) topography and b) friction (lateral force) of the composite surface. Images analysis on these images give a filling fraction of 1.65 10^13^ CNT/m^2^ on height image and 2.5 10^13^ CNT/m^2^ on friction image.

**SEM vs C-AFM imaging (Figure S5)**

Figure: High resolution C-AFM images obtained using platinum AFM tip. The image reveals a strong correlation between the C-AFM and SEM imaging; the high magnification current map shows the open top-CNT structure. Moreover, it is shown a gradient of conductivity between the inner-shells and the outmost shell of MWCNT. The outer shells appear less conductive which may be attributed to the CNT-epoxy-CNT tunneling junction.

1. K.L. Johnson, Contact Mechanics, Cambridge University Press, Cambridge, 1985. [↑](#footnote-ref-2)
